# Development and effects of advanced cardiac resuscitation nursing education program using web-based serious game: application of the IPO model

**DOI:** 10.1186/s12912-024-01871-7

**Published:** 2024-03-27

**Authors:** Gyuli Baek, Eunju Lee

**Affiliations:** 1 School of Nursing, University of Pittsburgh, Pittsburgh, PA USA; 2 Keimyung University College of Nursing, Daegu, South Korea

**Keywords:** Advanced cardiopulmonary resuscitation, Nursing education program, Web-based serious game, IPO model

## Abstract

**Background:**

The significant rise in cardiac arrest cases within hospitals, coupled with a low survival rate, poses a critical health issue. And in most situations, nurses are the first responders. To develop nursing students’ competencies in advanced cardiopulmonary resuscitation, systematic and repetitive learner-centered self-directed education that can promote the integration of knowledge and practice is necessary.

**Objectives:**

To develop an advanced cardiopulmonary resuscitation training program using a web-based serious game for nursing students and verifying its efficacy.

**Design:**

The program was developed based on the stages of analysis, design, development, implementation, and evaluation, and the Input Process Outcome Model of Serious Game Design formed the theoretical basis.

**Settings and participants:**

The research design employed a before-and-after non-equivalent control group, and data collection took place among 2nd and 3rd year nursing students at K University in D City, Korea, from March 2, 2023, to March 24, 2023.

**Methods:**

The program consisted of a 120-min video lecture, 30 min of a web-based serious game, 30-min of written self-reported debriefing, and individual feedback using a video conference system. The effectiveness of the program was measured for both groups using an 89-item structured questionnaire regarding knowledge, confidence in performance, problem-solving ability, and learning transfer expectations.

**Results:**

The program was effective in improving nursing students’ advanced cardiopulmonary knowledge, confidence in performance, problem-solving ability, and learning transfer expectation immediately after intervention.

**Conclusions:**

This program underscores the necessity of a new direction in nursing education, emphasizing learner-centered approaches, rather than the traditional focus on the mere transmission of basic knowledge and skills, to cultivate nurses with advanced cardiopulmonary resuscitation capabilities.

## Introduction

Today, despite the development of modern medicine and emergency medical systems, the rapid increase in cardiac arrest patients in hospitals and the low survival rate in this group is recognized as an important global health problem [1, 2]. Among medical personnel in hospitals, nurses account for 75% of first responders in the event of cardiac arrest, and nurses’ advanced cardiopulmonary resuscitation (ACPR) capabilities are directly related to patient survival [3, 4]. Therefore, systematic education should be implemented in undergraduate courses so that nursing students, as future nurses, can develop competencies for effectively performing ACPR [5].

Education to strengthen nursing students’ ACPR competencies should not only aim to improve basic knowledge and performance but also foster nurses’ confidence and independent problem-solving skills [6]. Further, through experiential learning that can trigger learners’ interest and engagement, rather than theory-based education, it is necessary to meet the expectations of learning transfer, which is the individual’s perception that learning content will lead to performance [7–9]..

Today’s nursing students develop problem-solving skills through trial and error, and seek clear rewards and feedback for their efforts [10]. They are digital natives, called so because of their familiarity with information and communication technology [11, 12]. Therefore, web-based serious games intentionally produced for educational purposes and operating on websites through Internet interfaces are attracting attention as an active learning method apt for today’s nursing students [13].

However, since web-based serious games in the nursing field are currently in the introduction stage there are only research using serious games for the purpose of simple interest and fun instead specific presentation of reward and achievement-based feedback elements based on design models and learning outcomes through game experiences [14, 15].. In addition, it is limited to simple skill education such as blood transfusion, urethral Catheterization, and management of tracheostomy tubes [16–18]. Therefore, it is necessary to develop a nursing education program based on a systematic design model to strengthen the ACPR competency of nursing students.

Garris et al.’s [19] Input Process Outcome Model of Serious Game Design [IPO] is a learning experience based on system feedback, and a model that clearly presents the process of creating learning outcomes through games by presenting a debriefing process that links knowledge and experience to learning outcomes [19, 20]. In the context of team-based emergency management, serious games for medical and nursing students are reported to be effective in improving teamwork and communication skills by providing indirect experience and debriefing opportunities pertaining to emergency situations otherwise difficult to experience through clinical practice [12]. Further, a serious game visualizing disaster situation and combining the game characteristics of the IPO model and the severity classification training content has been found to be effective in promoting nursing students’ severity classification knowledge, confidence in performance, and learning transfer [9].

Therefore, the web-based serious game to which the IPO model is applied is effective in strengthening the ACPR competency of nursing students by implementing realistic digital contents that are difficult to directly experience cardiac arrest situations and providing realistic experiential classes centered on immediate feedback and interaction. It can be used as an educational strategy. Accordingly, this study aims to develop and apply a web-based serious game-based ACPR nursing education program to test the effects of nursing students’ knowledge of advanced cardiac resuscitation, performance, confidence in the performance, problem-solving ability, and learning transfer expectations.

## Purpose

The purpose of this study is to develop an ACPR nursing education program using a web-based serious game for nursing students and verify its efficacy.

## Methods

### Design

**Fig. 1:**
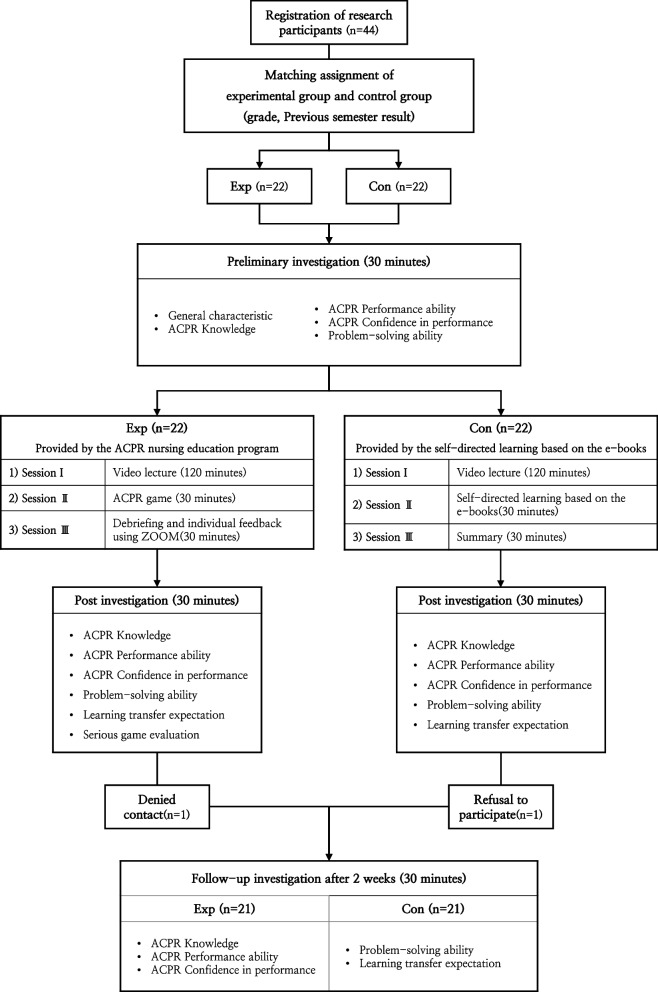
Research flow chart

This study employed a non-equivalent control group pretest-posttest design. The experimental group participated in an expert cardiac resuscitation nursing education program using a web-based serious game, and the control group received case-based self-directed learning using e-books. To verify the nursing education program’s efficacy, the knowledge, performance ability, confidence in performance, and problem-solving ability of ACPR were measured in three stages, with pretest, posttest and follow-up surveys, and the expectation of learning transfer was measured in the post-follow-up surveys. Figure 1 schematizes the overall flow of this study.

### Participants

The participants were second- and third-year nursing students attending K University located in D City in Korea, who understood the purpose and contents of the study and agreed to participate. The inclusion criteria were as follows: 1) students with no vision or hearing problems, 2) students who have not completed the cardiac arrest simulation class in school, and 3) students who have not obtained KALS or ACLS certificates. For sample size estimation, the G power version 3.1.9 (Heinrich Heine University, Dusseldorf, Germany) program was used. The number of samples required for the two-group repeated measure analysis of variance (ANOVA) was based on a simulation intervention study for nursing students [21]; the median effect size was 0.25, the power (1-β error probability) was 0.80, and the significance level was .05. Calculations revealed that a total of 34 samples were required; however, considering the dropout rate of 10–30% reported in previous studies using serious games and web-based simulations among nursing students [9, 22], the number of participants was calculated as 22 in each group considering a 20% dropout rate, for a total of 44 participants.

### Measures

#### ACPR knowledge

To evaluate ACPR knowledge, we used the ACPR knowledge tool developed by Chae and Choi [23] for nursing students, and adapted the tool by adding items on integrated treatment and communication after cardiac arrest with the original authors’ permission. Following the tool’s content validity assessments by two nursing professors and one nurse with 10 years of experience in the emergency room, we derived a content validity index (CVI) of 1.0. The tool consists of 20 questions, and each question has a four-point multiple choice format, with 1 point for the correct answer and 0 points for the wrong answer; the higher the score, the higher the ACPR knowledge. In the original study, the CVI of the tool was 0.8 or higher, and in this study, the reliability of the tool using the Kuder-Richardson Formula 20 (KR20) was found to be .82.

#### ACPR performance ability

To test ACPR performance ability, we used the ACPR performance ability tool developed by Choi [24]. The tool comprises 27 questions based on a five-point Likert scale (1 point = “very bad”; 5 points = “very good”); the higher the score, the higher the ACPR performance ability. In Choi’s [24] study, the Cronbach’s ⍺ was .98, and that in this study was .96.

#### ACPR confidence in the performance

To test ACPR confidence in performance, we used the ACPR confidence in the performance tool developed by Chae and Choi [23]. It comprises 10 questions indicating the degree of ACPR confidence in performance (1 point = “Not at all” and 10 points = “Very Much, Yes”); the higher the score, the higher the degree of ACPR confidence in performance. In Chae and Choi’s [23] study, the Cronbach’s ⍺ was .85, and in this study, it was .88.

#### Problem-solving ability

To assess problem-solving ability, we used the problem-solving ability tool developed by Lee [25] for high school students, and modified and supplemented it with the tool by Park and Woo [26] for college students. The tool comprises 25 questions based on a five-point Likert scale (1 point = “very rarely”; 5 points = “very often”); the higher the score, the higher the problem-solving ability. In Park and Woo’s [26] study, the Cronbach’s ⍺ was .90, and in this study, it was .89.

#### Expectation of learning transfer

We examined expectation of learning transfer using the tool developed by Lee [27] for organizational training, and modified and supplemented this with that developed by Lee [9] for nursing college students. The total comprises seven questions based on a five-point Likert scale (1 point = “Not at All”; 5 points = “Very Much, Yes”); the higher the score, the higher the expectation for learning transfer. The Cronbach’s ⍺ was .75 in Lee’s [27] study and .95 in Lee’s [9] study, while that in the current study was .90.

## Intervention

### ACPR nursing education program

The development and evaluation of nursing education programs in this study were conducted in the stages of analysis, design, development, execution, and evaluation based on the ADDIE model [28].

In the first analysis stage, prior research within the past 5 years, 2020 Korean CPR guidelines [29], and related book analysis were explored to analyze educational content and game characteristics. Next, two nurses, one nursing professor, and one gaming mobile professor working at a higher general hospital validated the tool (CVI of .80 or higher) to adopt educational content and game characteristics in the questionnaire (Table 1).

**Table 1:** Result of Content Validity of Expert Group for Draft Nursing Education Program

Item		Content	CVI
Educational Content	1. Cardiac arrest definition and causes	• Recognize symptoms and signs of cardiac arrest patients	0.8
	2. Basic CPR	• Reaction confirmation, rescue request • Chest compressions, artificial respiration • How to use the Patient Immobilization Brace^a^	0.8
	3. Cardiac arrest rhythm	• Cardiac arrest rhythm monitoring and pulse check (Ventricular fibrillation, pulseless ventricular tachycardia, asystole, pulseless electrical activity)	1.0
	4. Advanced Airway maintenance	• Establishing and maintaining the airway • Preparation and application of advanced airway maintenance	0.8
	5. Administering a defibrillator	• Defibrillator preparation and application	1.0
	6. Emergency medication	• Emergency medication selection and application • Preparation and administration of injectable medications^a^ • Administer intravenous injection^*^	1.0
	7. Hypothermia treatment and integrated treatment after recovery of spontaneous circulation	• Hypothermia treatment after restoration of spontaneous circulation • Integrated treatment after recovery of spontaneous circulation	0.8
Game Characteristics	1. Goal	• Providing reward items for mission performance • Character growth through item acquisition	1.0
	2. Rules	• Provide step-by-step missions • Acquire items and points during missions • Presentation of target score and earned score • Limited time offer • More about the limit timeout step • Game ends when additional learning is completed	0.8
	3. Competition	• Character growth comparison through game results feedback page • Game via step display window and item window	0.8
	4. Confidence	• Provides missions considering progressive difficulty • Provides additional learning about mission failure steps	0.6
	5. Control	• Learner-centered game operation through mission performance	0.2
	6. System feedback	• Item rewards for completing missions • Character growth by acquiring items • Correct and incorrect feedback • Using the game result feedback page Self-reported written debriefing • Individual feedback	0.8

^a^Items added after implementation of expert group content validity

In the second design stage, learning goals were set and design elements of the game characteristics and development environment were created. The learning goal of this program was set to “quickly identify cardiac arrest situations in hospitals and provide high-quality advanced cardiac resuscitation.” Next, a clinical case-based scenario for game design was created. Based on the format proposed by Jeffries and Rogers [30], the scenario was scripted by dividing it into time, rhythm process, patient mannequin behavior, expected intervention, and clues, and was written as a cardiac arrest situation of patients in general wards.

**Fig. 2:**
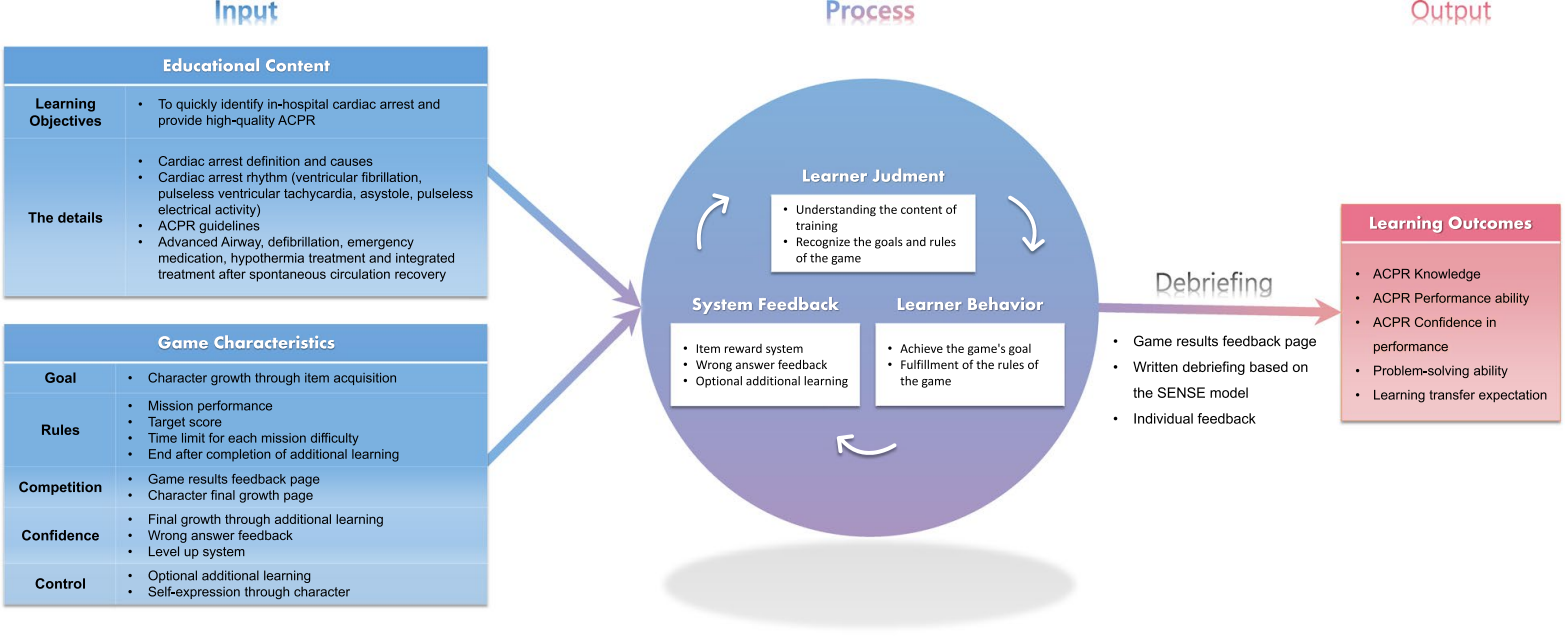
Theoretical framework of this stu

In the third stage of development, educational materials were produced and a system interface for ACPR games was established. A system interface was built to mount games on Amazon Web Services (Amazon Web Services [AWS], Seattle, USA) and connect to Google Form, which allows users to write self-reported debriefing after the game is over (Fig. 2). Next, the UI/UX of the game, screen composition and menu arrangements, and detailed content arrangement were performed in collaboration with 3D designers to create a user interface for ACPR games. The 3D designers and animators were then commissioned to produce characters and items to be inserted into ACPR games to perform 3D character modeling, texturing, and lighting.

In the fourth execution stage, a preliminary survey was conducted with three experts (two nurses working at a higher general hospital and a professor at a nursing university, certified by the American Heart Association or the Korea Cardiopulmonary Resuscitation Association) to evaluate the usability of ACPR games.

In the final evaluation stage, an appropriateness evaluation was conducted with three users (third-year nursing students) to finally complete the nursing education program. The user appropriateness evaluation of the ACPR game was conducted using the serious game evaluation questionnaire [31], and the total score was 96.3 ± 6.35.

### Data collection

#### Participants

Data collection was conducted from March 2, 2023 to March 24, 2023, involving second- and third-year nursing college students. Before collecting data, the study’s purpose and procedures were presented to the head of the relevant department, and approval was secured. Recruitment involved posting notices on school bulletin boards and social networks. After announcing the study’s purpose, duration, recruitment details, and progress specifics, applicants were accepted on a first-come, first-served basis. The process consisted of the pre-post-surveys and the follow-up. To ensure the homogeneity of the experimental group and the control group, participants with the same grades of the previous semester were paired and placed into two groups. Cooperation was requested to prevent exchanges; explanations on the study were provided and written consent was obtained. A total of 44 participants were recruited, 22 each in the experimental and control groups.

#### Pre-test

The pre-test, post-test, and then follow up of the experimental and control groups were conducted by online and were conducted individually on a date agreed upon in advance by the participants. During the pre-survey period, general characteristics, knowledge, performance ability, confidence in performance, and problem-solving ability were measured through an online questionnaire in both two groups. The time required to fill out the questionnaire was 20–30 min, and all 44 participants completed the questionnaire in advance.

#### Intervention

Immediately after the pre-survey was conducted, the experimental treatment was administered to both groups. The nursing education program of the experimental group consisted of a total of three sessions. In Session I, we presented a 120-min video lecture based on the research of Oh et al. [32] and provided the KALS curriculum. In Session II, the experimental group participated in the ACPR game, wherein learners cannot skip the contents during the game or move to the last screen, and learners must complete the game to be connected to the Google form for self-report written debriefing. The time required for intervention was 30 min. In Session III, debriefing based on the game driving result table and individual feedback using ZOOM were provided to the experimental group. The debriefing was conducted in five stages: Sharing-Expansion-Attention-Support-Expansion, according to the SENSE model of debriefing developed by Ko and Choi [33].

For the control group, 30 min of self-directed learning using e-books was implemented based on a web-based simulation intervention study [22], after the lecture on the same prior theory as the experimental group. Case-based scenarios were provided, and learners were allowed to choose the appropriate ACPR steps and nursing processes for their respective situations. Finally, a 30-min summary and organize time was provided.

#### Post-test

Immediately after the intervention was completed, the general characteristics of the experimental and control groups, and their knowledge, performance ability, confidence in performance, problem-solving ability, and learning transfer expectations, were measured through an online questionnaire. Moreover, to measure the effect of the developed ACPR game, a questionnaire was administered to the experimental group to evaluate the serious game.

#### Follow-up

The follow-up of the experimental and control groups was conducted through an online questionnaire by selecting a follow-up date 2 weeks after the intervention was completed. Among the 44 participants, one dropped out (refused to be contacted), and one did not participate in the follow-up survey; thus, the data of 42 participants were analyzed.

## Data analysis

The data were analyzed using IBM SPSS Statistic 27.0 (IBM Corp., Armonk, NY, USA). The homogeneity test of the two groups was analyzed using Chi-square test and Fisher’s exact test, the preliminary homogeneity test of dependent variables was analyzed using an independent t-test, and the effect test on dependent variables was verified by repeated measures ANOVA and an independent t-test.

## Ethical considerations

This study was conducted after obtaining approval from the bioethics committee of the institution to which the researcher belongs to consider the ethical aspects of the subject (IRB No. 40525–202,210-HR-057-03).

## Result

### Prior homogeneity test based on general characteristics and dependent variables

Most of the participants were female students, and their grades based on self-report were in the middle of the previous semester (Table 2). In the homogeneity test of the two groups, there were no general characteristics that showed a statistically significant difference between them. The preliminary homogeneity test with the dependent variables, knowledge, performance ability, confidence in performance, and problem-solving ability, revealed no statistically significant difference between the experimental and control groups across all variables, and homogeneity was secured.

**Table 2:** Prior homogeneity test about general characteristics and dependent variable (*N* = 42)

Variable	Category	Exp. (*n* = 21)	Con. (*n* = 21)	χ^2^, t	*p*
		*n*(%) or M ± SD			
Gender^*^	Male	1(4.7)	2(9.6)	0.31	1.000
	Female	20(95.3)	19(90.4)		
Grade^*^	2nd	16(76.2)	16(76.2)	0.00	1.000
	3nd	5(23.8)	5(23.8)		
Previous semester result ^*^	< 3.0	4(19.0)	4(19.0)	0.00	1.000
	3.0–3.5	6(28.6)	6(28.6)		
	3.5–4.0	9(42.8)	9(42.8)		
	4.0–4.5	2(9.6)	2(9.6)		
Major Satisfaction^*^	High	12(61.9)	15(71.5)	7.17	.099
	Average	6(28.6)	5(23.8)		
	Low	2(9.5)	1(4.7)		
ACPR Knowledge		52.14 ± 19.07	53.33 ± 13.54	0.23	.817
ACPR Performance Ability		2.39 ± 0.88	2.60 ± 0.64	0.87	.385
ACPR Confidence in Performance		3.50 ± 1.97	3.54 ± 1.69	0.06	.947
Problem-Solving Ability		3.35 ± 0.55	3.22 ± 0.42	−0.81	.420

* Fisher’s exact test

### Differences in ACPR knowledge, performance ability, confidence in performance, and problem-solving ability between the two groups before and after education

Table 3 shows that the experimental group’s ACPR knowledge was 52.14 ± 19.07 before intervention, 81.19 ± 8.50 after intervention, and 78.10 ± 8.87 2 weeks after intervention, which was higher than the control group results (53.33 ± 13.54 before, 71.42 ± 11.95 after, and 59.76 ± 11.00 after 2 weeks). Each group (*F* = 8.21, *p* = .007), each time point (*F* = 11.60, *p* < .001), and the interaction between time point and group all showed statistically significant differences (*F* = 44.09, *p* < .001). The experimental group’s ACPR performance ability was 2.39 ± 0.88 before intervention, 3.99 ± 0.63 after intervention, and 4.03 ± 0.56 2 weeks after intervention, higher than the control group (2.60 ± 0.64 before, 3.46 ± 0.61 after, and 3.34 ± 0.53 after 2 weeks). There was no significant difference between groups (*F* = 3.10, *p* = .086), but the time point (*F* = 248.33, *p* < .001) and the interaction between time point and group showed statistically significant differences (*F* = 28.91, *p* < .001).

**Table 3:** Differences in ACPR knowledge, performance ability, confidence in performance, and problem-solving ability between two groups before and after education (*N* = 42)

Variables	Exp. (*n* = 21)	Con. (*n* = 21)	t	p*	Categories	F	*p*†
	M ± SD						
**ACPR Knowledge**							
Pretest	52.14 ± 19.07	53.33 ± 13.54	0.23	.817	Group	8.21	.007
Posttest	81.19 ± 8.50	71.42 ± 11.95	−3.05	.004	Time	11.60	<.001
After 2 weeks	78.10 ± 8.87	59.76 ± 11.00	−5.94	<.001	Group*Time	44.09	<.001
**ACPR Performance Ability**							
Pretest	2.39 ± 0.88	2.60 ± 0.64	0.87	.385	Group	3.10	.086
Posttest	3.99 ± 0.63	3.46 ± 0.61	−2.73	.009	Time	248.33	<.001
After 2 weeks	4.03 ± 0.56	3.34 ± 0.53	−4.02	<.001	Group*Time	28.91	<.001
**ACPR Confidence in Performance**							
Pretest	3.50 ± 1.97	3.54 ± 1.69	0.06	.947	Group	199.30	<.001
Posttest	7.91 ± 1.34	5.84 ± 1.20	−5.24	<.001	Time	22.05	<.001
After 2 weeks	8.15 ± 1.13	5.79 ± 1.27	−6.35	<.001	Group*Time	13.95	<.001
**Problem-Solving Ability**							
Pretest	3.35 ± 0.55	3.22 ± 0.42	−0.81	.420	Group	23.88	<.001
Posttest	4.08 ± 0.44	3.32 ± 0.42	−5.60	<.001	Time	49.64	<.001
After 2 weeks	4.16 ± 0.39	3.23 ± 0.36	−7.96	<.001	Group*Time	39.86	<.001

*Exp.* Experimental group, *Cont.* Control group

* *P*-value of t-test between groups at each time point

† Repeated Measures ANOVA *p*-value between groups at each time point

The experimental group’s ACPR confidence in performance was 3.50 ± 1.97 before intervention, 7.91 ± 1.34 after intervention, and 8.15 ± 1.13 2 weeks after intervention, which was higher than the control group (3.54 ± 1.69 before, 5.84 ± 1.20 after, and 5.79 ± 1.27 after 2 weeks). Each group (*F* = 199.30, *p* < .001), time point (*F* = 22.05, *p* < .001), and the interaction between time point and group showed statistically significant differences (*F* = 13.95, *p* < .001). The problem-solving ability of the experimental group was 3.35 ± 0.55 before intervention, 4.08 ± 0.44 after intervention, and 4.16 ± 0.39 2 weeks after intervention, which was higher than the results of the control group (3.22 ± 0.42 before, 3.32 ± 0.42 after, and 3.23 ± 0.36 after 2 weeks). Statistically significant differences were noted by group (*F* = 23.88, *p* < .001), time point (*F* = 49.64 *p* < .001), and the interaction between time point and group (*F* = 39.86, *p* < .001).

### Differences in learning transfer expectation between the two groups after education

Table 4 shows that the learning transfer expectation in the experimental group was 4.84 ± 0.20 points post-survey and 4.87 ± 0.19 points 2 weeks after, which was higher than that of the control group (4.20 ± 0.48 points post-survey and 3.97 ± 0.47 points 2 weeks after), and each group (*F* = 51.57 *p* = .016), time point (*F* = 6.38 *p* < .001), and the interaction between time point and group (*F* = 11.75, *p* < .001) all showed statistically significant differences.

**Table 4:** Differences in learning transfer expectation two groups after education (*N* = 42)

Variables	Exp. (*n* = 21)	Con. (*n* = 21)	t	p*	Categories	F	*p*^†^
	M ± SD						
Posttest	4.84 ± 0.20	4.20 ± 0.48	−5.60	<.001	Group	51.57	.016
After 2 weeks	4.87 ± 0.19	3.97 ± 0.47	−8.06	<.001	Time	6.38	<.001
					Group*Time	11.75	<.001

*Exp.*, Experimental group, *Cont.* Control group

* *P*-value of t-test between groups at each time point

† Repeated Measures ANOVA *p*-value between groups at each time point

### ACPR game evaluation

Analyzing the evaluation of the experimental group on the ACPR game, we obtained the result of 3.24 ± 0.19 points out of 4 points. Among them, the teaching-learning goal and content item showed the highest score with 3.89 ± 0.17 points, while the technical item showed the lowest score with 3.66 ± 0.53 points.

## Discussion

In this study, a nursing education program was developed using the IPO model proposed by Garris et al. [19] as a theoretical framework. Further, a web-based serious game, centered on realistic digital content and immediate feedback and interaction, was used to improve learning transfer expectations by inducing learners’ interest. In the input stage of the IPO model, the nursing education program was designed by deriving the educational contents that should be included in the ACPR training through literature analysis and expert validity. Additionally, by including the selected educational content and the five-game characteristics of the IPO model’s goal, rules, competition, and confidence control, we tried to spark learners’ interest and enhance learning effects.

This study aimed to boost learning transfer expectations in nursing education by combining realistic digital content with the gamified features of the IPO model. Learning transfer expectations, a crucial link between educational content and performance, should be considered in designing effective nursing education programs [27]. To achieve this, using realistic digital content to engage learner interest is essential [9]. Building on prior research, the study employed strategies like first-person video shooting [34] and incorporating background music to create urgency [35]. Therefore the nursing education program was effective in enhancing learning transfer expectations. And also, In the follow-up, the experimental group’s expectations improved compared to the control group, indicating sustained program effectiveness. These findings align with the positive impact of the IPO model-based game on learning transfer expectations in nursing students [9]. However, it’s important to note that the measured expectations are subjective views on future learning transfer, not the direct application of training knowledge to work. Future research should conduct long-term and follow-up studies targeting nursing students, assessing the impact on work performance in real clinical settings to determine the effectiveness of learning transfer through gamified approaches.

The nursing education program in this study was effective in improving the knowledge of ACPR. At the two-week follow-up, the reduction in knowledge of the experimental group tended to be significantly lower than that of the control group. This is consistent with the research results that the IPO model-based triage serious game and the experiential game model-based transfusion nursing serious game are more effective in improving knowledge than traditional lectures [9, 18, 19, 36]. However, when simple game characteristics were used without the presentation of a design model, previous studies showed no effect of improving knowledge compared to traditional lectures [37]. Therefore, to develop an effective nursing education program, a structured approach based on a design model is required to achieve an appropriate balance between educational content and games [19, 20].

The nursing education program in this study was also effective in improving the performance ability of nursing students. Additionally, at two-week follow-up, unlike the control group, the experimental group’s performance ability improved. This was similar to the results of studies that showed that a serious game for nursing students with COVID-19 patients and a serious game on transfusion nursing for nurses had a positive effect on performance ability improvement [18, 37]. Based on previous studies, this study is considered to have improved performance ability because it provided realistic digital content, immediate feedback, and repeated performance [38]. However, the nursing education program in this study evaluated the performance ability of ACPR in a web-based virtual environment, and has limitations in that it did not provide an opportunity to perform more realistic nursing practice and did not conduct an objective evaluation of the same. Therefore, in the future, it is necessary to conduct research using various virtual reality motion recognition devices so that learners can directly perform ACPR in a serious game.

The nursing education program in this study was effective in improving nursing students’ confidence in performance. Further, at two-week follow-up, the confidence in performance of the control group decreased, while the experimental group showed improved results. This is similar to the results of previous studies showing that COVID-19-related nursing serious games, blood transfusion nursing serious games, and web-based nursing education programs for patients with acute heart disease are effective in improving confidence in performance and self-efficacy in nursing students [5, 18, 37]. Therefore, it is necessary to develop a nursing education program that can provide continuous clinical practice mastery and successful experience in nursing performance based on scenarios that reflect the clinical situation to improve nursing students’ confidence in performance.

Further, the nursing education program in this study was found to be effective in improving the problem-solving ability of nursing students. At follow-up, unlike the control group, the problem-solving ability of the experimental group improved. Based on the IPO model, this study provides a means to induce learner judgment and learner behavior in the game process stage, and presents a problem-solving method for learner behavior through system feedback, so that learners can independently identify and clarify problems in cardiac arrest situations [19, 20].. Additionally, after the game, self-reported written debriefing and individual feedback were provided to facilitate learners’ structural reflection and improve their problem-solving abilities. However, in the case of this study, there is a limitation in that the effect of interaction is lacking due to the lack of opportunities for open communication between colleagues who act as facilitators in the debriefing process. Therefore, in future research, it is suggested to add a group debriefing function in the form of real-time discussion through simultaneous access by learners to maximize interactions between learners.

### Implications for practice

This study developed a nursing education program based on the IPO model as a theoretical framework and implemented it with nursing students. Additionally, through systematic feedback and debriefing sessions, learners engaged in self-evaluation and reflection on the educational content. It was demonstrated to be effective in enhancing knowledge of ACPR, performance ability, confidence in performance, problem-solving ability, and learning transfer expectation. Thus, the nursing education program in this study can serve as a comprehensive ACPR competency enhancement program, facilitating the integration of theoretical knowledge and practical skills in managing cardiac arrest situation, which are often challenging to encounter in clinical practice. Furthermore, it can bolster the ACPR proficiency of nursing students, equipping them to conduct successful resuscitation maneuvers in clinical settings. Moreover, by extending its application to nurses across various clinical environments, this program holds promise in potentially improving the survival rates of cardiac arrest patients by ensuring the provision of high-quality professional resuscitative care.

### Limitations

First, since this study was conducted by within a single university, there are limitations in generalizing the research results. Second, in this study, a web-based serious game using digital contents was developed to provide learners with a realistic learning experience, but there are limitations in the part where interaction was not maximized through group debriefing through simultaneous access between learners. Third, this study measured the subjective expectation that learning transfer would occur, not the learning transfer that affects work by applying the contents learned through education to actual work, and the performance ability of ACPR in a web-based serious game. Therefore, there is a limitation in that it did not provide opportunities for actual nursing practice to learners and did not evaluate the effect on learning transfer and performance skills.

## Conclusions

This study developed an ACPR nursing education program using a web-based serious game for nursing students and confirmed its effectiveness. The nursing education program provided in this study was effective in improving the ACPR knowledge, performance ability, confidence in performance, problem-solving ability, and learning transfer expectation of nursing students. As a result of a follow-up survey after 2 weeks, it was confirmed that there was an effect on improving performance ability, confidence in performance, problem solving ability, and learning transfer expectation. Therefore, the nursing education program of this study presents a new direction for learner-centered education in nursing education in the twenty-first century, can be used as an effective educational intervention, and ultimately contributes to an opportunity to grow fostering nurses equipped with ACPR competency.

## Data Availability

The data that support the findings of this study are available on request from the corresponding author. The data are not publicly available due to privacy or ethical restrictions.
